# *IQCB1 (NPHP5)*-Retinopathy: Clinical and Genetic Characterization and Natural History

**DOI:** 10.1016/j.ajo.2024.03.009

**Published:** 2024-08

**Authors:** SAGNIK SEN, LORENZO FABOZZI, KAORU FUJINAMI, YU FUJINAMI-YOKOKAWA, GENEVIEVE A. WRIGHT, ANDREW WEBSTER, OMAR MAHROO, ANTHONY G. ROBSON, MICHALIS GEORGIOU, MICHEL MICHAELIDES

**Affiliations:** 1 Moorfields Eye Hospital (S.S, L.F., K.F., G.W., A.W., O.M., A.R., M.G., M.MM), London, United Kingdom; 2 UCL Institute of Ophthalmology (S.S., K.F., Y.F.-K., A.W., O.M., A.R., M.G., M.M.), University College London, London, United Kingdom; 3 Laboratory of Visual Physiology, Division of Vision Research (K.F., Y.F.-Y.), National Institute of Sensory Organs, NHO Tokyo Medical Center, Tokyo, Japan; 4 Department of Health Policy and Management (Y.F.-Y.), Keio University School of Medicine, Tokyo, Japan; 5 Jones Eye Institute (M.G.), University of Arkansas for Medical Sciences, Little Rock, Arkansas, USA

## Abstract

**Purpose:**

To describe the clinical and genetic features, and explore the natural history of retinopathy associated with *IQCB1* variants in children and adults with retinopathy.

**Design:**

Retrospective cohort study at a single tertiary care referral center.

**Methods:**

The study recruited 19 patients with retinopathy, harboring likely disease-causing variants in *IQCB1*. Demographic data and clinical presentation, best corrected visual acuity (BCVA), fundus appearance, optical coherence tomography (OCT) and autofluorescence features, electroretinography (ERG) and molecular genetics are reported.

**Results:**

Ten patients had best corrected visual acuity better than 1.0 LogMAR, and BCVA remained stable till the last review. Seven patients had a vision of hand movements or worse in at least one eye at presentation. There was no correlation found between age of onset and severity of vision loss. Nine patients (47.4%) had a diagnosis of end-stage renal failure at presentation. The other 10 patients (52.6%) had a diagnosis of non-syndromic *IQCB1*-retinopathy and maintained normal renal function until the last follow-up. The mean age at diagnosis of renal failure was 26.3 ±19.8 years. OCT showed ellipsoid zone (EZ) disruption with foveal sparing in 8/13 patients. All patients had stable OCT findings. Full-field ERGs in four adults revealed a severe cone-rod dystrophy and three children had extinguished ERGs. We identified 17 *IQCB1* variants, all predicted to cause loss of function.

**Conclusion:**

*IQCB1*-retinopathy is a severe early-onset cone-rod dystrophy. The dissociation between severely decreased retinal function and relative preservation of retinal structure over a wide age window makes the disease a candidate for gene therapy.

## INTRODUCTION

Leber congenital amaurosis/early-onset severe retinal degeneration (LCA/EOSRD) is a spectrum of inherited retinal degenerations (IRDs).^1^^,^^2^ LCA frequently leads to significant visual impairment from birth / early infancy, with minimal/absent electrophysiological responses, along with a variable degree of nystagmus, photophobia and oculodigital sign.^3^^,^^4^ LCA/EOSRD is most commonly inherited as an autosomal recessive disorder and is genetically heterogenous. More than 25 genes have been described to be disease-causing for 70% of LCA/EOSRD cases.

The *IQCB1* gene (IQ calmodulin-binding motif-containing protein-1; also known as *NPHP5*) is located on chromosome 3q13.33, with pathologic variants associated with Senior-Loken syndrome (SLS), a rare autosomal recessive condition involving renal dysfunction and LCA/EOSRD. The renal disease occurs in the form of nephronophthisis, presenting with symptoms such as polyuria, polydipsia, secondary enuresis, and anemia.^5^ Chronic renal disease usually slowly progresses to end-stage renal disease (ESRD). More than 20 genes have been described for nephronophthisis-related ciliopathies (NPHP), of which *NPHP1* homozygous deletions result in overall 21% of all NPHP cases; whereas the rest of the genes contribute to less than 3% each.^5^ Of these, *IQCB1/NPHP5,* has been termed the classical SLS gene, since pathogenic variants in *IQCB1/NPHP5* have been most characteristically associated with renal and retinal manifestations.^5^ *NPHP6/CEP290* gene is one of the most common causes of isolated LCA overall, without nephronophthisis. Previous studies have reported several disease-causing variants in *IQCB1/ NPHP5*, and these variants may be associated with SLS or isolated LCA/EOSRD.^7^, ^8^, ^9^

In the current study, we describe the clinical and genetic characteristics, explore phenotype-genotype correlations, the disease natural history, and the potential window for intervention in a large cohort of molecularly proven *IQCB1/NPHP5* patients.

## METHODS

This retrospective study protocol adhered to the tenets of the Declaration of Helsinki and received approval from the Moorfields Eye Hospital (MEH) ethics committee. Informed consent was obtained from all adult subjects, whereas informed consent and assent were obtained from parents and children (<15 years of age), respectively. All patients were seen and identified by medical retina specialists in the genetics/medical retina and pediatric retina clinics.

### CLINICAL EXAMINATION AND RETINAL IMAGING

All available clinical notes were reviewed. Visual acuity (VA), refraction, fundoscopy, and slit-lamp biomicroscopy findings were extracted. Age of onset was defined as the age at which the family first noticed any symptoms and sought medical care. Age seen was the age at which the patient was first seen at our referral center.

Best corrected logarithm of the minimum angle of resolution (LogMAR) visual acuity (BCVA) was assessed, monocularly, with the Early Treatment Diabetic Retinopathy Study (ETDRS) chart or using Snellen charts. Visual acuity in children was measured using Teller acuity cards. Where VA was measured using Snellen charts, the measurement was converted to LogMAR units for analysis. Refraction was undertaken by a specialist optometrist for both adults and children. Interocular symmetry in BCVA was defined as a difference of BCVA within 0.3 LogMAR between the two eyes.

Color fundus imaging was obtained by conventional 35-degree fundus imaging (Topcon Great Britain Ltd Berkshire, UK) or ultra-widefield (200-degree) confocal scanning laser imaging (Optos plc, Dunfermline, UK). Fundus autofluorescence (FAF) imaging was performed using 30-degree or 55- degree Spectralis (Heidelberg Engineering Ltd, Heidelberg, Germany), or Optos (Optos plc Dunfermline, UK) imaging. Spectral-domain optical coherence tomography (OCT) scans (Spectralis; Heidelberg Engineering Ltd) were used to assess cross-sectional and longitudinal structural changes.

### ELECTROPHYSIOLOGY

Pattern and full-field electroretinogram (PERG; ERG) testing was performed in 4 adults (ages 37-54 years), incorporating the International Society for Clinical Electrophysiology of Vision (ISCEV) standards^10^^,^^11^ using corneal recording electrodes. The PERG P50 component was used as a measure of macular function and dark-adapted (DA) and light-adapted (LA) ERGs were used to assess generalized rod and cone system function.^12^ The responses were compared with an age-matched control group.^13^ Additionally, flash ERGs were recorded from 3 young children (aged 6-18 months) using non-Ganzfeld flashes and lower eyelid skin electrodes, according to a shortened ERG protocol.^11^^,^^14^^,^^15^

### GENETIC ANALYSIS

Genomic DNA was isolated from peripheral blood leukocytes (Gentra Puregene Blood Extraction Kit; Qiagen, Venlo, Netherlands). A combination of Sanger direct sequencing and next-generation sequencing, including a panel of retinal dystrophy genes,^16^ whole exome sequencing,^17^ and whole genome sequencing,^18^ was used to identify variants in *IQCB1*.

All recruited patients were reassessed for their detected *IQCB1* variants (*IQCB1*: Refseq Reference: NM_001319107.2; Emsembl transcript ID: ENST00000310864.11; UniProtKB: Q15051). Sequence variant nomenclature was obtained according to the guidelines of the Human Genome Variation Society (HGVS) by using Mutalyzer 2.0.^19^ Classification of all detected variants was also performed based on the guidelines of the American College of Medical Genetics and Genomics (ACMG).^20^^,^^21^

*In silico* molecular modeling was conducted. Minor allele frequency for the identified variants in the general population was assessed in the Genome Aggregation Database (gnomAD, version 4.0) datasets. The population data and general coverage by whole exome sequence were also provided with the gnomAD database. General prediction scores were further calculated using MutationTaster, FATHMM, CADD, and REVEL. Functional prediction was performed employing SIFT, PROVEAN, and Polyphen 2. Human splicing finder 3.0 was applied for splicing defects prediction. Mammalian (PhyloP30way and PhastCons30way) and vertebrate (PhyloP100way and PhastCons100way) conservation were also investigated. The previously reported variants were surveyed with the HGMD database and ClinVar database (accessed on January 2023). The evolutionary conservation was assessed by multiple alignments of species of the *IQCB1* gene sequence using the Clustal Omega program (https://www.ebi.ac.uk/Tools/msa/clustalo/).

## RESULTS

### DEMOGRAPHICS

Nineteen patients (9 male, 10 female) from 17 pedigrees, were identified with pathogenic *IQCB1* variants. At first presentation in our tertiary referral center (MEH, London, UK), the mean age (±SD) was 25.0 (±23.4) years (range; 4 months to 70 years), with an average follow-up period (±SD) of 7.3 ± 5.8 years (range; 2 to 18 years). Three patients had a history of consanguinity in parental marriages. Seven out of the 17 pedigrees had more than one disease-affected member (siblings). Data from two pairs of siblings were available. Demographics and clinical information for all patients are presented in Table 1.

**Table 1:** Demographic and clinical features of study cohort

Family ID	Patient ID	Consanguinity	Onset of symptoms	Family history	Sex	Renal failure	Age at presentation (y)	Visual acuity at presentation	Current Age	Current Visual acuity	Phenotype
29690	P1	No	since birth	None	F	Yes*	32	HM, HM	34	HM, HM	LCA
20067	P2	No	1 year	Sister	F	Yes*	72	HM, HM	79	HM, HM	LCA
25739	P3	No	since birth	None	M	No	3	NLP	6	NLP	LCA
24625	P4	No	1 year	None	M	Yes*	50	6/24, 6/24	55	6/36, 6/36	LCA/EOSRD
21142	P5	No	early childhood	Brother	F	No	6	6/36, 6/36	13	6/75, 6/60	LCA
15195	P6	No	since birth	None	F	Yes**	4	CF, CF	21	HM, HM	LCA
23193	P7	Yes	50 years	Siblings	M	No	60	6/12, 6/36	65	6/12, 4/60	CRD
23832	P8	Yes	since birth	None	F	No	23	LP, LP	-	-	LCA
23194	P9	No	early childhood	None	F	Yes	42	6/12,6/12	44	6/12,6/12	CRD
16927	P10	No	since birth	None	M	No	3	1.7 BEO	17	6/190, 6/151	LCA
18993	P11	No	since birth	None	F	Yes	1	6/60 BEO	13	6/180	LCA
23006	P12	No	since birth	None	M	No	1	LP, LP	7	NLP, NLP	LCA
25933	P13A	No	39 years age	Sibling of P13B	F	Yes	41	6/18, 6/12	46	6/15, 6/15	CRD
25933	P13B	No	31 years age	Sibling of P13A	F	Yes*	47	6/36, 6/36	49	2/60, 4/60	CRD
28832	P14	Yes	early childhood	None	M	Yes*	25	6/96, LP	27	HM, HM	LCA/EOSRD
29862	P15	No	since birth	None	M	No	Birth	LP, LP	2	LP, LP	LCA
16850	P16A	No	4 years	Sibling of P16B	M	No	18	6/18, 6/18	36	3/60, 3/60	CRD
16850	P16B	No	6 years	Sibling of P16A	F	No	19	6/36, 6/18	35	6/18, 6/18	CRD
25264	P17	No	30 years	None	M	Yes*	40	3/60, 3/60	-	-	EORD

y; years, F; female, M; male, HM; Hand motion, CF; counting fingers, LP; light perception, NLP; no light perception, LCA; Leber's Congenital Amaurosis, BEO; both eyes open, EOSRD; Early Onset Retinal Dystrophy, CRD; Cone-rod Dystrophy. * Patient received renal transplant. ** Patient listed for renal transplant.

### CLINICAL PRESENTATION AND FOLLOW-UP

The clinical diagnosis was LCA/EOSRD in 13 patients (68.4%) and cone-rod dystrophy in 6 (31.6%). Of the patients who were diagnosed with LCA/EOSRD, seven patients had onset from birth and six patients since early childhood. The most common symptom at presentation was loss of vision (100%), followed by difficulty in daylight (n = 15/19, 78.9%) and reduced color vision (n = 14/19, 73.6%). Other symptoms were not being able to follow light (n = 3/19, 15.8%) or no response to light (n = 2/19, 10.5%). The seven patients (36.8%) with congenital disease had nystagmus at presentation. Two patients (10.5%) with oculodigital sign developed keratoconus. The average follow-up in the cohort was 7.3 ± 5.8 years.

Nine patients (47.4%) were diagnosed with renal failure, either at presentation or after further investigation. The mean age at diagnosis of renal failure was 26.3 ± 19.8 years (median, 23 years; range, 10-70 years). The diagnosis of SLS was established for those patients after genetic testing. P1 was detected with small kidneys at birth. Six patients (n = 6/9, 67%) had already undergone renal transplantation at presentation to MEH and P6 was listed for renal transplant. The average age ±mean at kidney transplantation was 28.6 ± 8.3 years (median = 29 years; range, 15-41 years). The remaining patients had a diagnosis of non-syndromic LCA/EOSRD or CORD, and maintained normal renal function at presentation until last follow-up (range of follow-up = 2-18 years). The average age ±SD of patients with no renal dysfunction was 24.8 ± 20 years (median, 20 years; range, 2-44 years).

Based on the age of onset of symptoms and/or presentation and BCVA, we identified the following 4 patterns: (1) onset from birth or early childhood (first 5 years of life) with very poor vision (n = 10, 52.6%), (2) onset from early childhood (first 5 years of life) with relatively good vision (n = 2, 10.5%), (3) presentation in adulthood with relatively good vision (n = 3, 15.8%), and (4) presentation in adulthood with poor vision (n = 1, 5.2%). Refraction data was available in 8 patients, with 7 being hyperopic and 1 myopic.

### VISUAL ACUITY

The BCVA at presentation ranged from 0.3 LogMAR to no perception of light. Most patients (n = 15/19, 78.9%) had symmetric BCVA at presentation. Nine patients (9/19, 47.4%) had BCVA at presentation better than or equal to 1.0 LogMAR (6/60 Snellen) in their better seeing eye, and ten (10/19, 52.6%) had BCVA worse than 1.0 LogMAR (6/60 Snellen). Seven (36.8%) had a vision of hand movements or worse in at least one eye at presentation. Four (21%) patients (P7, P11, P13B, P14) had a drop in BCVA from presentation after an average follow-up of 5.4 ± 4.7 years (median, 3.7 years; range, 2-12 years). Five (26.3%) either had relatively stable BCVA or had a worsening by 1 or 2 Snellen lines (P4, P5, P7, P9, P13A) in one eye only. Table 1 presents the visual acuity at baseline and last follow-up for all patients.

### FUNDUS APPEARANCE

**Figure 1:**
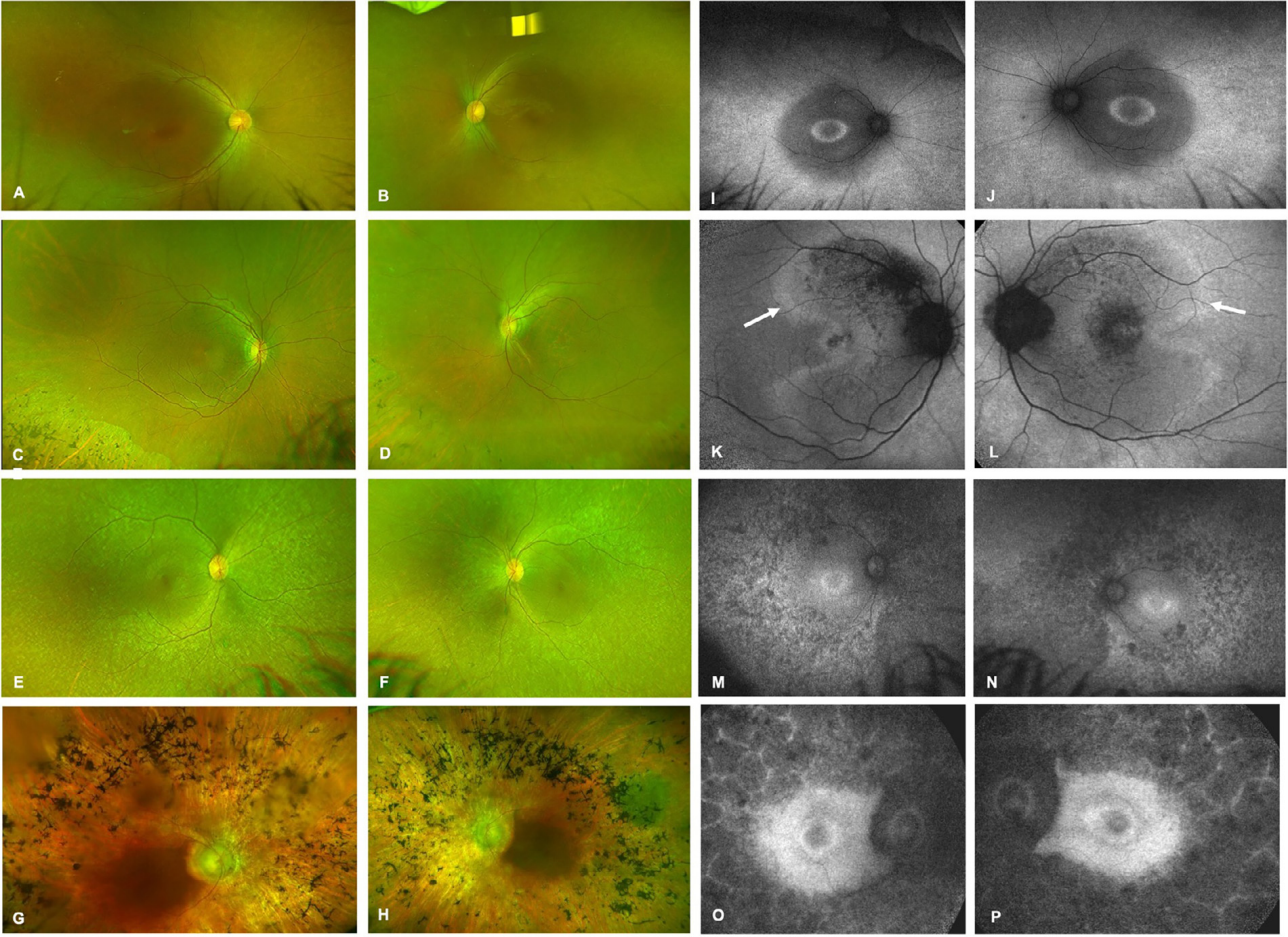
Color Fundus and Fundus Autofluorescence Imaging in *IQCB1*-Retinopathy. (A and B) Images from a 13-year-old female patient who developed symptoms from birth and a diagnosis of LCA, showing relatively normal appearing macula with few patchy peripheral pigmentary changes. (C and D) Images from a 65-year-old male patient who developed symptoms at 50 years age, with a diagnosis of cone rod dystrophy, showing relatively normal macula with peripheral lobular chorioretinal atrophy with bone spicules. (E and F) Images from a 23-year-old female patient and (G and H) from a 79-year-old female patient, both of whom developed symptoms at birth with a diagnosis of LCA, showing extensive macular and peripheral hypopigmentation with bone spicules. (I and J) Images showing presence of a perifoveal ring and perimacular ring. (K and L) Images showing presence of localized hypoautofluorescent patches, and a perimacular ring, as well as high density perimacular curvilinear arcs marked with white arrows. (M-P) Images showing extensive peripheral hypoautofluorescence with confluence of patches in O and P to form a lobular pattern of autofluorescence.

**FIGURE 2:**
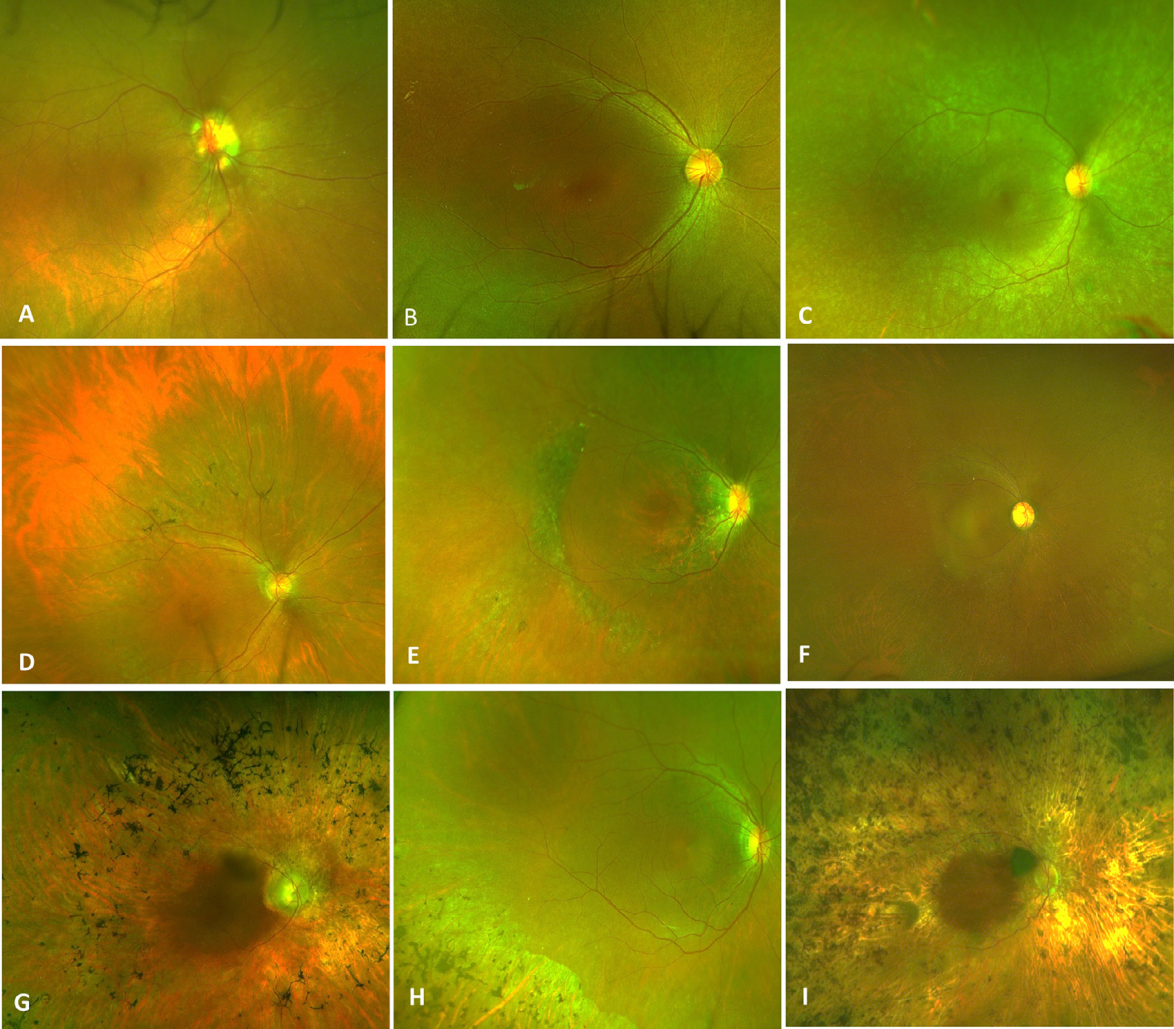
Fundus Appearance of Patient with *IQCB1*-Retinopathy by Age. Color fundus photographs of the right eye retina arranged in ascending order of age at the final visit; (A, B) in 13-year-old patients followed by 23 years (C), 34 years (D), 44 years (E), 46 years (F), 55 years (G), 65 years (H) and 79 years (I) of age. A greater extent of retinal pigmentary changes is observed in older individuals.

**FIGURE 3:**
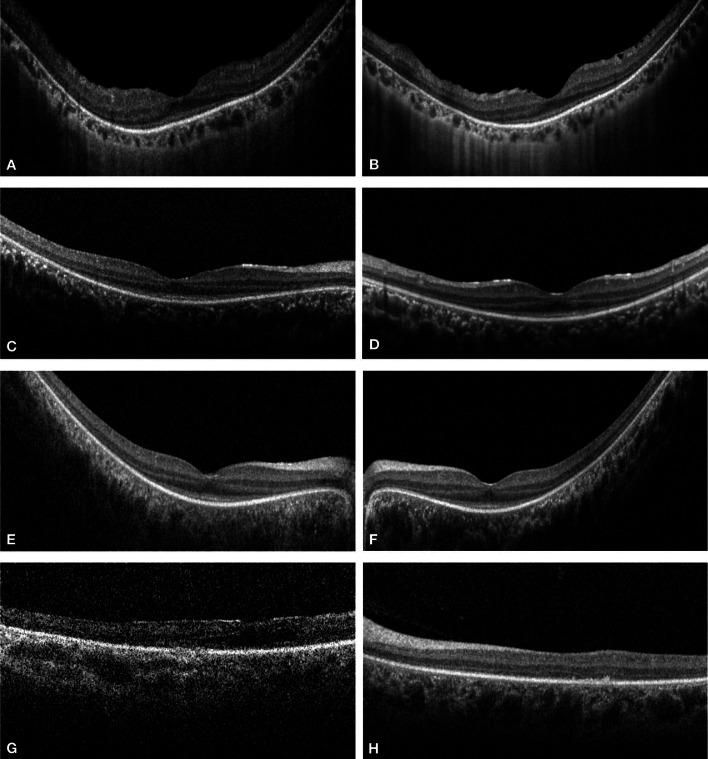
Changes on SD-OCT Imaging in *IQCB1*-Retinopathy. (A and B): The right and left eye scans from a 44-year-old female patient showing preserved foveal ellipsoid zone. (C and D) The right and left eye scans from a 6-year-old female patient showing preserved foveal ellipsoid zone at presentation. (E and F) Scans from the same patient as in (C and D) after a period of 7 years showing no significant progression of changes in the ellipsoid zone loss. (G and H): The right and left eye scans from a 34-year-old female patient showing extensive loss of ellipsoid zone.

Color fundus photographs were available for 13 patients (68.4%). We observed the following types of fundus appearances with varying degrees of hypopigmentation and hyperpigmentation: (1) relatively normal appearing macula with patchy peripheral pigmentary changes (Figure 1, A and B), (2) relatively normal macula with peripheral lobular chorioretinal atrophy with bone spicules (Figure 1, C and D), and (3) extensive macular and peripheral hypopigmentation with or without bone spicules (Figure 1, E-H). Similar fundus findings were noted in unrelated patients with nonoverlapping *IQCB1* gene variants. Longitudinal imaging after an average follow-up of 7.3 ± 5.8 years did not demonstrate a significant change in fundus phenotype. However, cross-sectional analysis of the cohort did show more prominent pigmentary changes in older individuals (Figure 2).

### FUNDUS AUTOFLUORESCENCE IMAGING

FAF images were captured for 13 patients (68.4%) and all showed a high degree of inter-ocular symmetry. The most common patterns noted were mid-peripheral hypoautofluorescence, limited to a few areas (Figure 1, I-L) or extensive involving all quadrants (Figure 1, M-P), a perifoveal ring of increased signal (Figure 1, I,J, M-P) and high density perimacular curvilinear arcs (Figure 1, K and L). Patients P2 and P4 demonstrated extensive paracentral hypoautofluorescent lesions, arranged in a cobblestone fashion, with thin hyperautofluorescent intervening areas (Figure 1, O and P). Longitudinal images from these patients after an average follow-up ±SD of 7.3 ± 5.8 years did not show any significant change in pattern.

### OPTICAL COHERENCE TOMOGRAPHY

OCT scans were available for 13 patients (68.4%) (Figure 3). The ellipsoid zone showed some degree of disruption in all patients, with relative sparing at the fovea seen in 8/13 patients (61.5%). Patients with no OCT scans had very poor vision or were too young to cooperate for imaging. Longitudinal imaging was available for 6 patients, however there was no evidence of qualitative progression after a mean follow-up time of 5.0 ± 2.6 years.

### ELECTROPHYSIOLOGICAL FINDINGS

**FIGURE 4:**
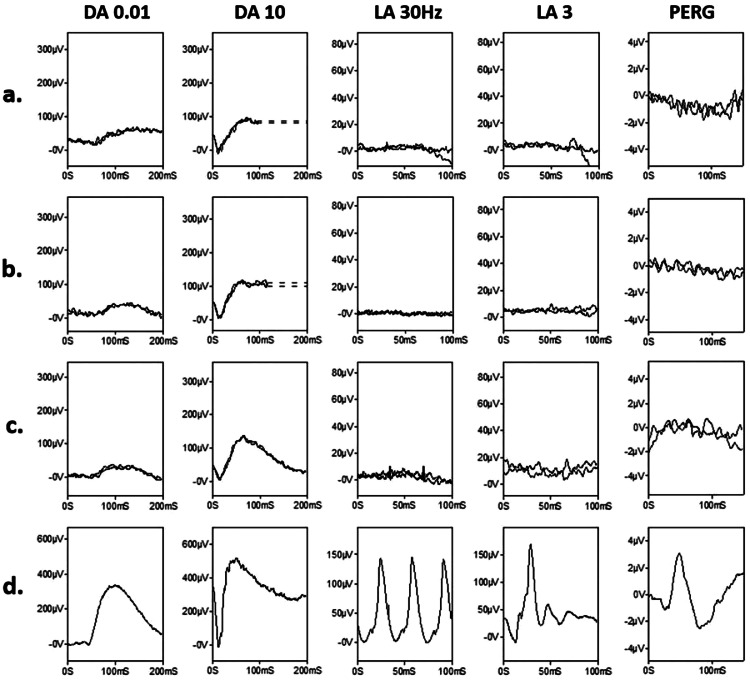
Electroretinography Findings in *IQCB1*-Retinopathy. Full-field and pattern ERGs from patients P9 (a: 37 years), P15A (b; 41 years) and P15B (c; 38 years). Representative control (d; “normal”) recordings are shown for comparison. Data are shown for the right eyes only, as all showed a high degree of inter-ocular symmetry. Scaling of patient traces is increased compared with the control recordings, to better illustrate waveforms. Patient traces are superimposed to demonstrate reproducibility. Broken lines replace blink artefacts for clarity. In all patients there is ERG evidence of a severe cone-rod dystrophy. DA 10 ERG b-wave peak times are delayed by 1 ms (a) 3 ms (b) and 5 ms (c) compared with age-matched controls values. Pattern ERG P50 is undetectable in all cases, consistent with severe macular involvement.

International standard full-field ERGs recorded in 4 adults, showed a high degree of inter-ocular symmetry based on the amplitudes of the DA 0.01 and DA 10 ERG a- and b-waves (slope 0.97; r^2^ = 0.98). The LA 30Hz (flicker) ERG, LA3 (single flash) ERG, and PERG P50 were undetectable in all 4 cases and DA ERGs were abnormal but detectable (examples are shown in Figure 4). The DA 0.01 ERGs were reduced by 75% to 81%, DA10 ERG a- waves by 63% to 80%, and b-waves by 53% to 63% compared with the lower limit of the reference range. DA 10 ERG a-waves showed borderline (n = 2) or mild delay (by 1.5 ms or 3 ms) and b-waves showed a delay in all 4 individuals (by 1, 3, 5, or 12 ms), worst in the oldest subject tested (54 years). Scotopic and photopic ERGs in three children, tested with lower eyelid skin electrodes, were undetectable bilaterally.

### MOLECULAR GENETICS

Whole genome sequencing was applied in 6 families (35.2%; P1, P2, P4, P6, P13A/B. P17), and whole exome sequencing was used in 4 families (23.5%; P2, P5, P11, P16A/B), with virtual panel filtering for retina-associated genes. Targeted exome next generation sequencing capture panels were employed in 6 families (P3, P7, P9, P12, P14, P15). These comprehensive genetic analyses were performed in combination with Sanger direct sequencing. Only Sanger direct sequencing was done in 2 families (11.7%; P8, P10).

All patients had two disease-causing variants; ten patients were compound heterozygotes and nine had homozygous variants. We identified 17 rare variants, with three not previously reported. The predicted effect of the variants in our cohort was determined according to HGVS (NCBI), with the majority of the variants being stop gained (8/17, 47.1%), followed by frameshift (6/17, 35.3%), one exon deletion (1/17, 5.9%), splice donor variant (1/17, 5.9%) and splice acceptor variant (1/17, 5.9%). A pathogenic criterion (PVS1) was met in all 17 variants with loss-of-function. Supplementary Table 1 presents the detailed assessment of the detected variants.

**FIGURE 5:**
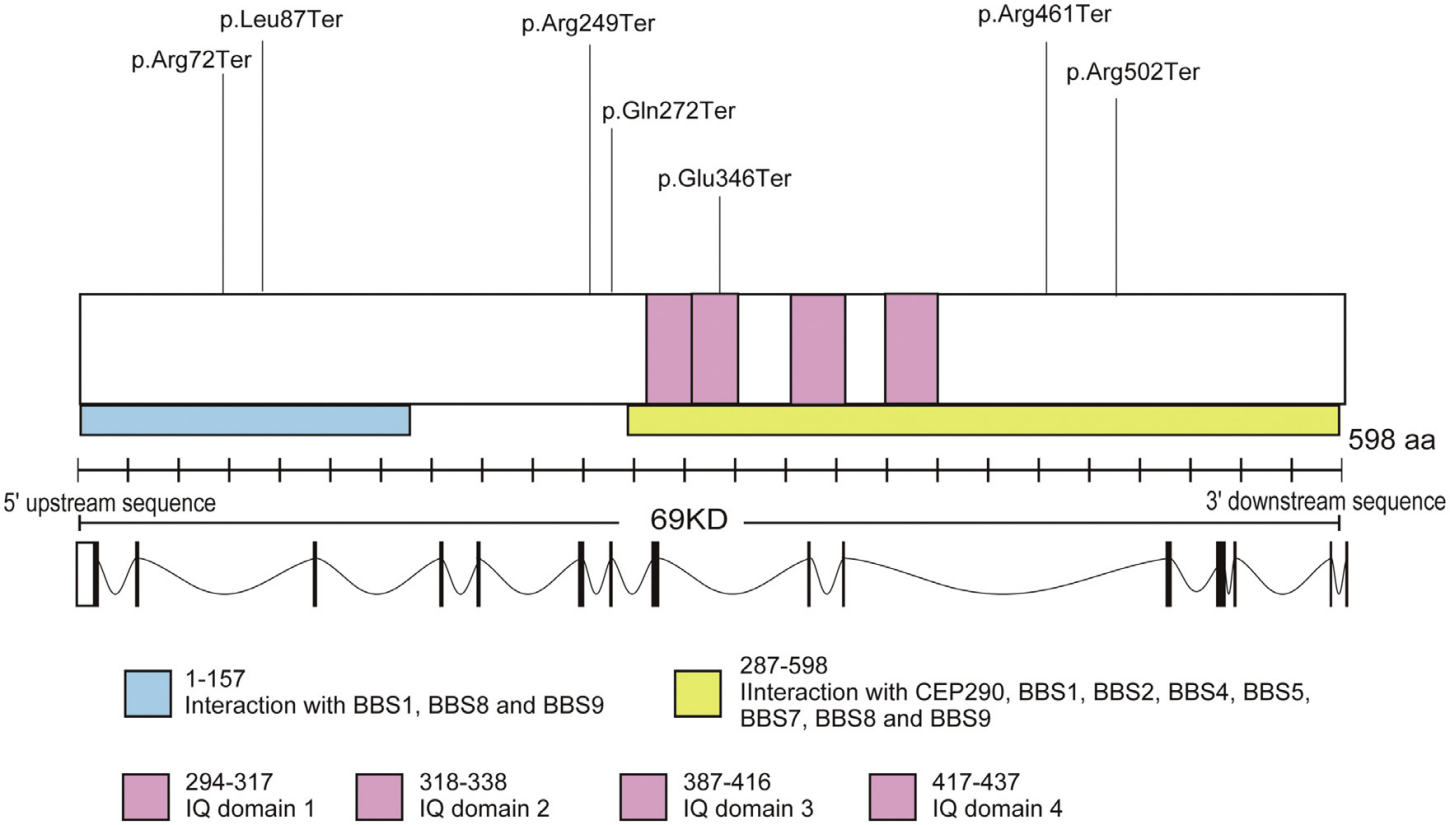
*IQCB1* gene and variants identified in the current cohort. The schematic shows a representation of the location of variants in the *IQCB1* gene along with their relation to the binding domains of the gene. It also shows known interactions of the different regions of the protein with BBSome and CEP290. All 17 variants were truncating variants predicted to show loss-of-function.

The most common variant in the cohort was c.1518_1519del (3/34 alleles, 8.8%). We summarize the genetic results from this cohort in Table 2 and Figure 5. The evolutionary conservation with multiple alignments of species of the *IQCB1* gene sequence is presented in Supplementary Figure 1.

**Table 2:** Summary of variants identified in the study cohort

Family ID	ID	Allele 1			Allele 2		
		DNA variant	Protein variant	Coding impact	DNA variant	Protein variant	Coding impact
29690	P1	c.897-900dup	p.Ile301LeufsTer42	frameshift variant	c.814C>T	pGln272Ter	stop gained
20067	P2	c.214C>T	p.Arg72Ter	stop gained	c.424-425del	p.Phe142ProfsTer5	frameshift variant
25739	P3	c.1036G>T	p.Glu346Ter	stop gained	c.1518-1519del	p.His506GInfsTer13	frameshift variant
24625	P4	c.1518 1519del	p.His506GInfsTer13	frameshift variant	c.897-900dup	p.Ile301LeufsTer42	frameshift variant
21142	P5	c.814C>T	pGln272Ter	stop gained	c.1504C>T	p.Arg502Ter	stop gained
15195	P6	c.260T>G	p.Leu87Ter	stop gained	c.1036G>T	p.Glu346Ter	frameshift variant
23193	P7	c.700 701del	p.Leu234ThrfsTer5	frameshift variant	Homozygous		
23832	P8	c.488-1G>A	-	splice acceptor variant	Homozygous		
23194	P9	del Exon 9	-	NA	del Exon 9	-	-
16927	P10	c.424-425del	p.Phe142ProfsTer5	frameshift variant	Homozygous		
18993	P11	c.1278+1G>A	-	splice donor variant	c.1381C>T	p.Arg461Ter	stop gained
23006	P12	c.1518-1519delCA	p.His506GInfsTer13	frameshift variant	c.424-425del	p.Phe142ProfsTer5	frameshift variant
25933	P13A/B	c.745A>T	p.Arg249Ter	stop gained	Homozygous		
28832	P14	c.1363C>T	p.Arg455Ter	stop gained	Homozygous		
29862	P15	c.1381C>T	p.Arg461Ter	stop gained	c.1381C>T	p.Arg461Ter	stop gained
16850	P16A/B	c.745A>T	p.Arg249Ter	stop gained	c.825-828del	p.Arg275SerfsTer6	frameshift variant
25264	P17	c.224-225del	p.Phe142PhefsTer5	frameshift variant	Homozygous

### GENOTYPE-PHENOTYPE ASSOCIATIONS

No specific correlations between genotypes and specific phenotypic patterns were identified. Of the 9 patients with renal phenotype, 3 (33.3%) had a retinal phenotype of CORD. All except P4 and P13A had very poor VA at presentation. Out of the rest 10 patients with no renal dysfunction, 3 (30%) had a phenotype of CORD. All patients with CORD had milder retinal pigmentary changes. We have summarized the clinical and genetic findings in patients with the attenuated phenotype of isolated retinal degeneration in Supplementary Table 2.

All variants identified in the current study (n = 17) span the entire protein and are predicted to have complete loss-of-function. Some of the disease-causing sequence variants are located in region 1-257 (variants p.Arg72Ter, p.Leu87Ter), and in region 287-598 (p.Glu346Ter, p.Arg461Ter, p.Arg502Ter). p.Glu346Ter is located inside the IQ domain 2. Variants p.Arg249Ter and p.Gln272Ter are located close to region 287-598. There was no difference between patients with LCA/EOSRD compared to those with CORD in terms of the variant type/location. On comparing the sequence variants in patients with and without renal disease, we noted an overlap of variants in both groups, suggesting that there is no clear distinction between these groups genotypically.

## DISCUSSION

In the current cohort of 19 patients, we describe 17 pathogenic *IQCB1* variants in patients with retinal dystrophy, with or without renal disease. The study makes observations regarding the variability in clinical presentation and the age of onset of retinal and renal disease. This is the largest such cohort of SLS patients with characterization of the variants. Retinal function phenotypes are comprehensively described, including details relating to disease progression. A significant proportion of patients either presented with or progressed to having severe vision loss, although a few maintained good vision until last follow-up. Thirteen patients had LCA/EOSRD, including children with undetectable ERGs, in keeping with a severe photoreceptor dystrophy. Adult patients had electrophysiological evidence of severe cone-rod dystrophy.

All patients with SLS eventually develop retinal degeneration and renal failure, commonly in their first to third decade.^5^ SLS should be kept in the list of differentials in young patients presenting with visual deterioration and renal dysfunction. Most patients with renal dysfunction will require renal transplantation, and the condition does not recur in the transplant.^6^ Patients presenting with retinal disease can have undiagnosed renal dysfunction and an important implication of our study is that a prompt evaluation of renal function is necessary in such patients. Patients with renal disease developed this at a median age of 23 years and needed a transplant at a median age of 29 years. In the current cohort, patients developed advanced renal disease at a wide range of ages; from 10 years and up to 70 years of age; the second patient had still not undergone a transplant at 79 years of age, on her last follow-up.

Although SLS has been previously described as being associated commonly with juvenile nephronophthisis,^22^, ^23^, ^24^ which is the most common age of presentation of nephronophthisis,^25^ authors also have described late onset renal disease in SLS patients,^26^, ^27^, ^28^, ^29^ where advanced renal disease develops during the fifth or sixth decade. Those without renal dysfunction in the current cohort had a median age of 20 years; with the oldest patient being 65 years of age. A significant proportion of our cohort did not have renal disease at presentation and did not develop this during follow-up. Considering the wide range of age of onset of advanced renal disease, our data is not a true reflection of the absolute prevalence of renal disease in the cohort, since patients with isolated retinopathy may develop kidney disease anytime in their lifetime. Detection of kidney disease in such asymptomatic patients depends on a high index of suspicion and the extent of clinical testing they undergo. However, this study highlights the importance of offering testing for renal disease and surveillance of renal function in patients of LCA/EORD/CORD who test positive for *IQCB1* variants, and thereby reiterates the importance of offering genetic testing to this cohort of patients at clinical presentation.

Seventeen *IQCB1* variants, including three novel variants (c.224_225del, c.700_701del, and deletion of exon 9) were detected. All 17 truncating variants spanning the entire protein are predicted to have complete loss-of-function. Two variants with canonical splice site alterations (c.1278+1G>A and c.488-1G>A) were identified, both of which have been previously detected in LCA patients.^31^^,^^32^ We found no clear genotypic distinction between patients with or without renal disease. In keeping with the renal disease, the retinal phenotype may also show significant variability in the age of onset of symptoms ranging from birth to the 5th decade in our cohort; with variability also seen on multimodal retinal imaging. Presence of an increased signal perifoveal ring on autofluorescence imaging was a common finding. An increased signal ring was also observed in the mid-periphery in some patients. Previously authors have described a lobular appearance of hypopigmented lesions in the periphery.^30^ In this cohort, we noted minimal peripheral changes early in the disease, with greater extent of pigmentary changes with age (Figure 2). Interestingly, all the patients with the attenuated phenotype showed milder affection of fundus, with only mild peripheral pigmentary changes, although there was no specific correlation with the renal phenotype. Phenotypic variability in patients may be explained by environmental modifiers, or moreover, there may be other genetic modifiers that act to affect the pathogenicity of the diseased allele.^33^ We found no VUS that can explain a modifier effect. However, we have limited data access to the full genomic data in our study; thus, further analyses with more detailed data resources focusing on modifier factors could clarify genotype-phenotype correlations based on the genome level, instead of the variants level of this study.

Adults who had electrophysiological testing had evidence of severe loss of cone system function with moderate generalized rod photoreceptor involvement, and in children undetectable ERGs were consistent with a severe photoreceptor dystrophy involving cones and rods. Although not a specific or diagnostic finding, it is noted that the severe loss of cone function (undetectable LA ERGs) in the adult patients with only moderate rod involvement is somewhat unusual for a CORD, and may suggest *IQCB1*-retinopathy as a differential. The findings in children suggest severe early onset disease, although this may not be reflected in the VA, likely due to relative preservation of the outer retina in the central macula in some patients. The latter finding is contrary to other forms of LCA where extensive photoreceptor loss is seen early including the macula. Some patients with minimal clinically detectable retinal changes could have severe ERG abnormality, highlighting the importance of objective electrophysiological evaluation and functional phenotyping. A limitation of this study is the availability of ERG from a small subset of patients, which limits the power of conclusions, as well the use of skin electrodes and non-Ganzfeld flashes rather than a full protocol in children.

The FDA approval of gene therapy for *RPE65*-associated LCA has triggered significant interest in exploring gene therapy for other IRDs.^34^^,^^35^ *IQCB1*-retinopathy is a target for gene therapy.^36^^,^^37^ Recently an AAV-virus vector mediated *IQCB1* gene augmentation therapy in a canine model has shown to improve photoreceptor morphology and visual function^36^ in the form of recovery of rod and cone-mediated ERG responses. The safety, efficacy, and different doses of four therapeutic vectors with dog or human *IQCB1* transgenes, regulated by IRBP or G protein-coupled receptor protein kinase 1 (GRK1) promoters, were explored. Detailed functional and structural evaluation of patients with *IQCB1*-retinopathy can further elucidate the natural history of the disease and identify endpoints for upcoming trials.^38^^,^^39^

Relative preservation of the EZ suggests that these patients may have a potential benefit from gene therapies for photoreceptor rescue, with the possibility of preserving or restoring central function, irrespective of the severity of peripheral retinal dysfunction. The extent of preservation of the EZ along with age and certain baseline function metrics, may be important criteria for participant stratification for clinical trials. Outcome measures need to cover functional and structural parameters, as well as patient perspective (patient reported outcomes). That might include EZ preservation VA and contrast sensitivity improvement, mobility testing, ERG, as well as quality of life questionnaires. The anatomical window for intervention appears relatively wide, with preservation of retinal structure till late in life. Phase 1/2 trial duration for short term results will likely need to be in the range of 1 to 2 years, to explore safety and efficacy signals, since effectiveness could be focused on functional improvement rather than halting/slowing the degeneration.

There are certain inherent limitations of the study. Our institute being a stand-alone eye hospital does not have direct access to assessments for renal function testing undertaken at other units. Moreover, the total number of images available or the quality of imaging due to low vision do not allow further quantitative analysis of ellipsoid zone integrity. Further, this is a retrospective study, hence there is a lack of uniformity in functional evaluation of the patients.

In the current report, we identified dissociation of structure and function, and potentially a target for intervention in this group of patients. The disease can present with or without renal involvement. This study suggests that the *IQCB1* gene should be screened in all patients with LCA/EOSRD or a severe cone-rod dystrophy, and all patients with identified variants should have renal evaluation.

## CRediT authorship contribution statement

**SAGNIK SEN:** Writing – review & editing, Writing – original draft, Project administration, Methodology, Investigation, Funding acquisition, Conceptualization. **LORENZO FABOZZI:** Funding acquisition, Formal analysis, Data curation, Conceptualization. **KAORU FUJINAMI:** Formal analysis, Conceptualization. **YU FUJINAMI-YOKOKAWA:** Formal analysis. **GENEVIEVE A. WRIGHT:** Data curation. **ANDREW WEBSTER:** Investigation, Data curation, Conceptualization. **OMAR MAHROO:** Supervision, Methodology, Investigation. **ANTHONY G. ROBSON:** Methodology, Formal analysis, Data curation. **MICHALIS GEORGIOU:** Methodology, Investigation, Funding acquisition, Formal analysis, Data curation. **MICHEL MICHAELIDES:** Writing – review & editing, Writing – original draft, Formal analysis, Data curation, Conceptualization.

## Appendix Supplementary materials

Image, application 1

Image, application 2

Image, application 3
