# Supplementary material for: IQCB1 (NPHP5)-Retinopathy: Clinical and Genetic Characterization and Natural History
Source: Am J Ophthalmol. 2024 Aug;264:205–15. doi: 10.1016/j.ajo.2024.03.009 (PMC11752837; doi:10.1016/j.ajo.2024.03.009)
Supplement: Supplementary file 1 [file mmc1.docx]

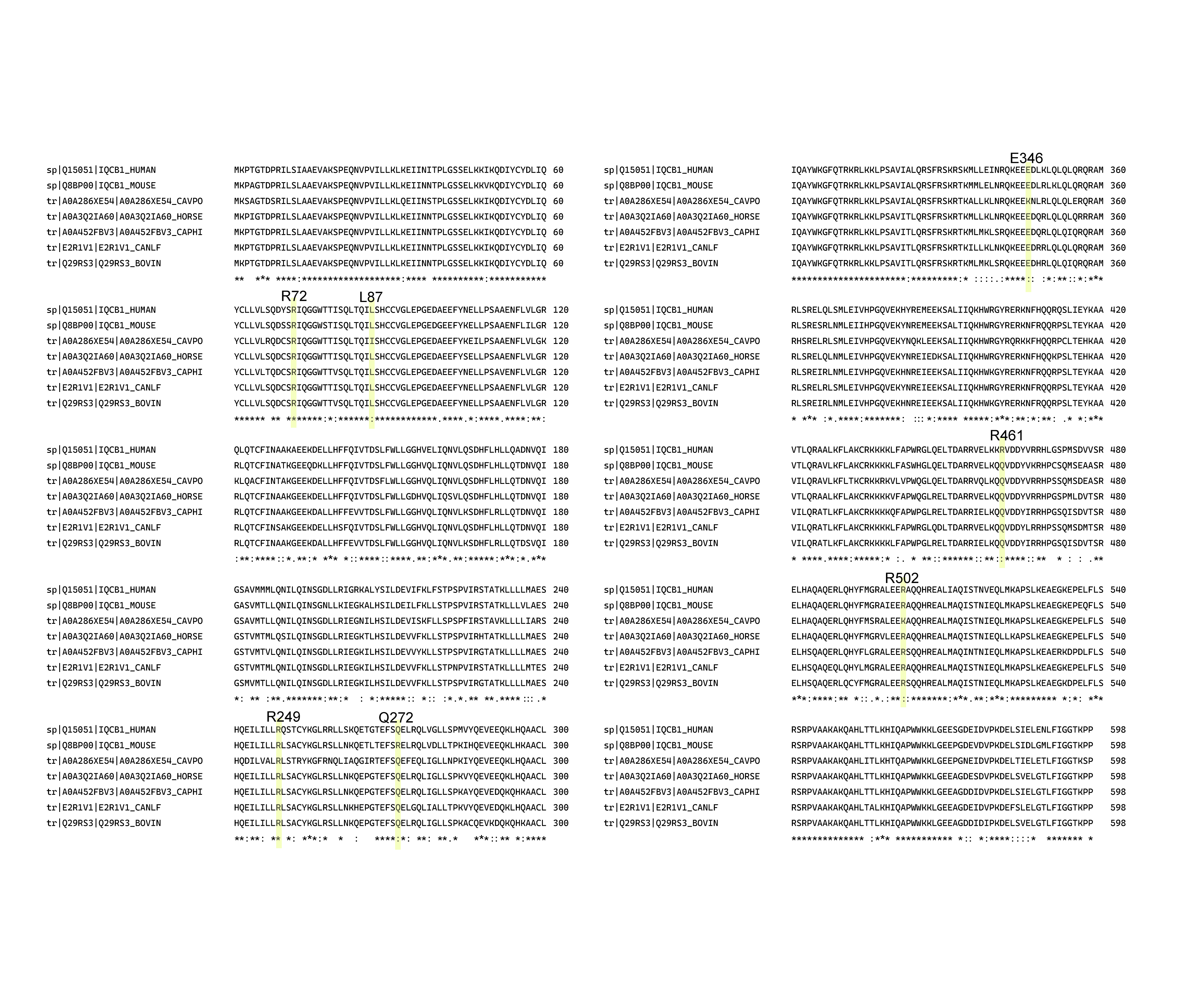


**Supplemental Figure 1**. Evolutionary conservation of the IQCB1 gene. Multiple alignments of seven species of the IQCB1 gene are demonstrated. The alignment was performed with the Clustal Omega program (https://www.ebi.ac.uk/Tools/msa/clustalo/), and the amino acid-sequence alignment was numbered in accordance with the Homo sapiens IQCB1 sequence (ENST00000310864.11). The locations of detected variants are highlighted with a yellow background. An asterisk indicates high conservation across the seven species.
