# Supplementary material for: IQCB1 (NPHP5)-Retinopathy: Clinical and Genetic Characterization and Natural History
Source: Am J Ophthalmol. 2024 Aug;264:205–15. doi: 10.1016/j.ajo.2024.03.009 (PMC11752837; doi:10.1016/j.ajo.2024.03.009)
Supplement: Supplementary file 3 [file mmc3.docx]

**Supplementary Table 2: Summary of patients with attenuated phenotype (isolated retinal disease) identified in the study cohort**

| **ID** | **Onset of symptoms (age in years)** | **Gender** | **Final Visual acuity** | **Allele 1** | | | **Allele 2** | |
| --- | --- | --- | --- | --- | --- | --- | --- | --- |
|  |  |  |  | **DNA variant** | **Protein variant** | | **DNA variant** | **Protein variant** |
| P3 | since birth | M | No response to light | c.1036G>T | p.Glu346Ter | | c.1518_1519del | p.His506GInfsTer13 |
| P5 | early childhood | F | 6/75, 6/60 | c.814C>T | pGln272Ter | | c.1504C>T | p.Arg502Ter |
| P7 | 50 years | M | 6/12, 4/60 | c.700_701del | p.Leu234ThrfsTer5 | | **Homozygous** | |
| P8 | since birth | F | NA | c.488-1G>A | - | | **Homozygous** | |
| P10 | since birth | M | 6/190, 6/151 | c.424_425del | p.Phe142ProfsTer5 | | **Homozygous** | |
| P12 | since birth | M | no appreciable response to light | c.1518_1519delCA | p.His506GInfsTer13 | | c.424_425del | p.Phe142ProfsTer5 |
| P15 | early childhood | M | not fixing/following light | c.1381C>T | p.Arg461Ter | | **Homozygous** | |
| P16A | 4 years | M | 3/60, 3/60 | c.745A>T | p.Arg249Ter | | c.825_828del | p.Arg275SerfsTer6 |
| P16B | 6 years | F | 6/18, 6/18 | c.745A>T | | p.Arg249Ter | c.825_828del | p.Arg275SerfsTer6 |
